# A Dispatch Screening Tool to Identify Patients at High Risk for COVID-19 in the Prehospital Setting

**DOI:** 10.5811/westjem.2021.8.52563

**Published:** 2021-10-27

**Authors:** Amy Albright, Karen Gross, Michael Hunter, Laurel O’Connor

**Affiliations:** University of Massachusetts Medical School, Department of Emergency Medicine, Worcester, Massachusetts

## Abstract

**Introduction:**

Emergency medical services (EMS) dispatchers have made efforts to determine whether patients are high risk for coronavirus disease 2019 (COVID-19) so that appropriate personal protective equipment (PPE) can be donned. A screening tool is valuable as the healthcare community balances protection of medical personnel and conservation of PPE. There is little existing literature on the efficacy of prehospital COVID-19 screening tools. The objective of this study was to determine the positive and negative predictive value of an emergency infectious disease surveillance tool for detecting COVID-19 patients and the impact of positive screening on PPE usage.

**Methods:**

This study was a retrospective chart review of prehospital care reports and hospital electronic health records. We abstracted records for all 911 calls to an urban EMS from March 1–July 31, 2020 that had a documented positive screen for COVID-19 and/or had a positive COVID-19 test. The dispatch screen solicited information regarding travel, sick contacts, and high-risk symptoms. We reviewed charts to determine dispatch-screening results, the outcome of patients’ COVID-19 testing, and documentation of crew fidelity to PPE guidelines.

**Results:**

The sample size was 263. The rate of positive COVID-19 tests for all-comers in the state of Massachusetts was 2.0%. The dispatch screen had a sensitivity of 74.9% (confidence interval [CI], 69.21–80.03) and a specificity of 67.7% (CI, 66.91–68.50). The positive predictive value was 4.5% (CI, 4.17–4.80), and the negative predictive value was 99.3% (CI, 99.09–99.40). The most common symptom that triggered a positive screen was shortness of breath (51.5% of calls). The most common high-risk population identified was skilled nursing facility patients (19.5%), but most positive tests did not belong to a high-risk population (58.1%). The EMS personnel were documented as wearing full PPE for the patient in 55.7% of encounters, not wearing PPE in 8.0% of encounters, and not documented in 27.9% of encounters.

**Conclusion:**

This dispatch-screening questionnaire has a high negative predictive value but moderate sensitivity and therefore should be used with some caution to guide EMS crews in their PPE usage. Clinical judgment is still essential and may supersede screening status.

## INTRODUCTION

In the midst of the severe acute respiratory syndrome-coronavirus disease 2019 (SARS-COVID-19) pandemic, there is an unprecedented need to screen for infectious disease in real time. Identification of patients at high risk for COVID-19 infection is essential in the setting of high infection rates, particularly to balance the need to conserve personal protective equipment (PPE) and ensure healthcare provider safety. Emergency medical services (EMS) personnel are at particularly high risk for exposures. They have less information and fewer resources to screen and test patients than their hospital-based counterparts. Thus, for EMS personnel the importance of PPE is paramount. However, there are several challenges to ensuring adequate protection due to concern for the EMS workers’ PPE fidelity and PPE conservation. Development of a screening tool that allows for detection of those most at risk for COVID-19 infection will aid the delicate balance of safety and conservation.

Literature is sparse on the efficacy of existing screening tools, and none evaluates the tools used by EMS dispatchers. Most dispatch-screening tools have not been studied for previous epidemic infectious diseases. Some screeners have been used to evaluate patients for COVID-19 infection in different clinical settings.^1^,^2^ Many of these published tools have shown utility but require findings such as imaging or laboratory testing, which are not available in the prehospital setting.^3^,^4^ Screeners in questionnaire format, including symptomatic surveillance and questions pertaining to high-risk exposures, have been used but not prospectively validated and are known to lead to high rates of false positives.^5^

To optimize safety in the EMS setting, a highly efficacious screening tool must have high sensitivity and a very high negative predictive value (NPV) to allow for high levels of confidence when deciding not to don full PPE. This tool should also be easy to administer, simple, and brief.^6^ The objective of this study was to determine the efficacy of an infectious disease surveillance tool for detecting patients who test positive for COVID-19 and the impact of positive screening on PPE utilization. Primary outcomes were the positive (PPV) and negative predictive values of the dispatch-screening tool. The secondary outcomes included PPE fidelity, PPE documentation, most common positive screening question, and the special populations most commonly positive for COVID-19.

## METHODS

**Figure 1:**
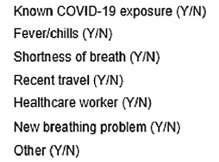
Dispatch screening questions.* *Yes to any question results in positive screen. *Y*, yes; *N*, no; *COVID-19*, coronavirus 2019.

This study was a retrospective chart review of prehospital care reports (PCR) and hospital electronic health records (EHR). We collected data from 911 calls placed between March 8–July 31, 2020 to an urban ambulance service serving a large, tertiary care center. We abstracted data from all 911 utilizations where the emergency medical dispatcher (EMD) documented the administration of a standardized screening tool. The instrument of interest used in this study was the Emerging Infectious Disease Surveillance Tool from the International Academies of Emergency Dispatch.^7^ A positive dispatch screen includes a “yes” to any of the questions on the included questionnaire. If the screen could not be completed, it was documented as an assumed positive. The contents of the instrument are depicted in Figure 1.

Metrics of interest included the question that triggered a positive screen, inclusion in a special population, and documentation of PPE use. The hospital EHR was reviewed for all patients who had a positive dispatch screen and their clinical course, including the results of their COVID-19 testing, and was abstracted successfully for all transported patients. The assay used for COVID-19 at the receiving hospitals was the Roche Cobas 6800 SARS-CoV-2 test (Roche Diagnostics, Basel, Switzerland) a highly sensitive duel-target, high-output polymerase chain reaction assay.^8^ Additionally, we queried the PCR and EHR for patients who had a negative dispatcher screen but ultimately tested positive for COVID-19, and abstracted their data. The institutional review board at the sponsoring institution approved this study.

## RESULTS

The ambulance service of interest transported 13,399 patients during the study period. A total of 4,329 patients had a positive COVID-19 EMD screen and 9,070 calls screened negative. In total, 263 patients had a positive COVID-19 test. Of those with a positive test, 197 had a positive EMD screen (74.9%, n = 197). Characteristics of the COVID-19 positive patients and fidelity of EMS personnel to PPE are described in Table 1. The prevalence of COVID-19 in the community of interest averaged 1.98% over the study period.

The sensitivity of the EMD screen was 74.9% (confidence interval [CI], 69.21–80.03) and the specificity was 67.71% (CI, 66.91–68.50). The screen’s PPV was 4.48% (CI, 4.17–4.80) and its NPV was 99.26% (CI, 99.09–99.40). When the screener’s performance was analyzed after excluding all instances where it could not be performed or was incomplete, its sensitivity was 70.93% (CI, 64.55–76.74) and its specificity was 67.68% (CI, 66.88–68.47). In this analysis, the PPV was 8.62% (CI, 7.96–9.33) and its NPV was 98.19% (CI, 97.79–98.52).

## DISCUSSION

This dispatch-screening questionnaire used by one institution’s EMS service is a useful initial tool to evaluate for patients at high risk of COVID-19 infection. It is short and simple, and evaluates enough metrics to achieve a NPV of 99.27%. However, its utility is limited by its sensitivity; the screen failed to detect one in four COVID-19-positive patients. Therefore, it must be used with caution and EMS agencies must consider their local disease prevalence and PPE availability when determining an appropriate interpretation of the screener’s efficacy. Ultimately, it may be prudent to don full airborne PPE for all EMS responses during a high-prevalence time such as a pandemic.

Despite its limitations, the screener has some utility in alerting crews to their highest risk patients. In many clinical settings, only patients who screen positive for specific symptoms are immediately placed on airborne precautions and some COVID-19-positive patients go undetected until they receive a positive test. The screeners help decrease, but do not eliminate, the number of high-risk exposures. The prehospital screener performs a similar function as a risk-mitigation strategy if complete PPE for every encounter is not feasible for EMS services.

Data on prior screening tools are scarce. One study, examining a questionnaire for travelers, found that screening tools missed up to half of infections.^9^ It is understandable that the questionnaire screening persons activating EMS has a lower rate of false negatives than a questionnaire for travelers, as participants seeking medical care are more likely to be symptomatic. The question that resulted in a positive screen was distributed across several responses and, therefore, it appears unlikely that any given question could be definitively eliminated.

The most common special population was residents of nursing facility (19.5%, n = 48). Other special populations much less commonly had positive tests and thus had minimal effect on the study including STEMI, stroke or trauma patient (n = 8, 3.3%), or drug/alcohol-related calls (n = 22, 8.0%). These populations were included because it was difficult to perform an effective prehospital screen on these patients due to altered mental status or critical illness; however, these patients rarely had positive COVID-19 tests. When such patients were excluded, however, the overall sensitivity of the screener was slightly decreased.

The use of PPE was not documented in 27.9% (n = 74) of PCRs for participants with positive screens. Without prospective study, however, it is difficult to analyze for PPE fidelity. This is an important shortcoming in documentation, as in the absence of documentation of PPE use in the PCR, follow-up from a patient’s positive COVID test becomes resource intensive. Without adequate documentation, time and resources may be spent contacting and quarantining personnel who actually may have been protected properly at the time of exposure.

## LIMITATIONS

This study was retrospective and relies on the documentation of dispatcher and EMS crews with regard to fidelity to the screener as well as PPE utilization. The prevalence of COVID-19 during the study period was low, which improved the NPV of the screener. The setting most likely facilitated relatively high sensitivity, as most participants were symptomatic and seeking emergency medical care. The language barriers faced by dispatchers responding to a highly diverse area may have also limited specificity of the screen; the screen was performed in English and a translator was used only when available. If a translator was not available then the screen was defaulted positive.

## CONCLUSION

Further study should be aimed at identifying the highest value screening questions so as to shorten the screening tool and increase sensitivity. Ambulance dispatch data as an early warning system for public levels of influenza-like illnesses and acute respiratory infections have been used as a public health tool in some cities, and data from this dispatch screener could potentially be used in a similar fashion.^10^–^16^ This study demonstrated that the described screening tool is a valuable instrument to evaluate for patients at high risk of being COVID-19 positive but should be used with caution to make decisions regarding use of personal protective equipment.

## Figures and Tables

**Table 1:** Characteristics of coronavirus-2019 positive patients (N = 263).

Positive screen (n, %)	
Yes	197 (74.9)
No	66 (25.1)
COVID test used (n, %)	
Rapid	100 (41.0)
PCR	144 (59.0)
Positive question (n, %)	
Known COVID19 contact	62 (32.0)
Fever/chills	54 (27.8)
Cough	54 (27.8)
Shortness of breath	100 (51.5)
Recent travel	0 (0.0)
Healthcare worker	5 (2.6)
Other breathing problem	5 (2.6)
Special population (n, %)	
Homeless	25 (10.2)
Skilled nursing facility	48 (19.5)
Drug/alcohol use	22 (8.9)
Trauma/STEMI/stroke	8 (3.3)
None	143 (58.2)
EMS PPE worn (n, %)	
Yes, full (N95, gown, gloves)	146 (55.7)
Partial	22 (8.4)
No	21 (8.0)
Not documented	74 (27.9)

*COVID-19*, coronavirus disease 2019; *PCR*, polymerase chain reaction assay; *STEMI*, ST-elevation myocardial infarction; *EMS*, emergency medical services; *PPE*, personal protective equipment.
